# Kre-Alkalyn® supplementation does not promote greater changes in muscle creatine content, body composition, or training adaptations in comparison to creatine monohydrate

**DOI:** 10.1186/1550-2783-9-S1-P11

**Published:** 2012-11-19

**Authors:** AR Jagim, Jonathan M Oliver, A Sanchez, Elfego Galvan, James Fluckey, S Reichman, S Talcott, K Kelly, C Meininger, Christopher Rasmussen, Richard B Kreider

**Affiliations:** 1 Department of Health and Kinesiology, Exercise and Sport Nutrition Laboratory, Texas A&M University, College Station, TX 77843-4243, USA

## Background

Creatine monohydrate (CrM) has been proven to be the most effective form of creatine and is considered the gold standard. However, a number of different forms of creatine have been purported to be more efficacious than CrM. The purpose of this study was to determine if a pH balanced form of creatine (Kre-Alkayn® (KA), *All American Pharmaceutical*, *Billings*, *MT*, *USA*) that has been purported to promote greater creatine retention and training adaptations with less side effects is more efficacious than CrM ingestion.

## Methods

In a double-blind manner, 36 resistance trained participants (20.2±2 yrs, 181±7 cm, 82±12 kg, 14.7±5 % body fat) were randomly assigned to supplement their diet with CrM (*Creapure®*, *AlzChem AG*, *Germany*) for 28-days (20 g/d for 7-d, 5 g/d for 21-d), an equivalent amount of KA as a high dose supplement (KA-H), or the manufacturer’s recommended dose of KA (1.5 g/d for 28-d, KA-L). Participants were asked to maintain their current training programs and record all workouts. Muscle biopsies from the vastus lateralis, fasting blood samples, body weight, DEXA determined body composition, 1RM bench press and leg press, and Wingate Anaerobic Capacity (WAC) tests were performed at 0, 7, and 28-days. Data were analyzed by MANOVA with repeated measures and are presented as mean ± SD changes from baseline after 7 and 28-d, respectively.

## Results

Muscle free creatine content increased in all groups over time (1.7±22 and 10.2±23 mmol/kg DW, p=0.03) with no significant differences among groups (KA-L –3.3±19.3, 0.53±22; KA-H 1±12.8, 9.1±23; CrM 8.2±32, 22.3±28 mmol/kg DW, p=0.19). In percentage terms, free creatine muscle content significantly increased over time (10.7±41, 29±46%, p= 0.003) with no differences observed among groups (KA-L -5.9±35, 11.9±40; KA-H 6.2±29, 27.3±49; CrM 34.6±50, 50.4±45%, p=0.10). Bodyweight increased in all groups over time (0.9±1.9, 1.42±2.5 kg, p<0.01) with no significant differences among groups (KA-L 0.7±0.83, 0.9±1.6; KA-H 1.7±2.9, 2.3±3.7; CrM 0.56±1.1, 1.1±1.4 kg, p=0.29). Fat-free mass significantly increased over time for all groups (0.67±0.9, 0.89±1.2 kg, p<0.01) with no differences among groups (KA-L 0.42±1.2, 0.37±1.3; KA-H 0.96±0.9, 1.2±1.4; CrM 0.6±0.8, 1.1±0.9 kg, p=0.16). Body fat percent decreased over time (-0.28±1, -0.22±1.4 %, p=0.42) for all groups with no differences among groups (KA-L -0.04±1.3, 0.15±1.2; KA-H -0.3±0.7, -0.31±1.6; CrM -0.5±0.9, -0.5±1.4 %, p=0.35). There was a significant increase in 1RM for bench press in all groups over time (8.1±9.7 kg, p<0.01) with no differences between groups (KA-L 7.1±3; KA-H 7.3±15; CrM 10±8 kg, p=0.73). There was no significant change in leg press 1RM (p=0.33). Total work performed on the WAC test increased in all groups over time (-69±1,030, 552±1,361 J, p=0.003) with no differences among groups (KA-L -278±676, 64±1,287; KA-H 412±1,041, 842±1,369; CrM -301±1,224, 775±1,463 J, p=0.27).

## Conclusions

Neither manufacturers recommended doses or equivalent loading doses of KA promoted greater changes in muscle creatine content, body composition, strength, or anaerobic capacity than CrM. These findings do not support claims that KA is a more efficacious form of creatine.

